# Large-scale geological structures of the Egyptian Nubian Shield

**DOI:** 10.1038/s41598-023-29008-x

**Published:** 2023-02-02

**Authors:** Zakaria Hamimi, Ahmed M. Eldosouky, Wael Hagag, Samir Z. Kamh

**Affiliations:** 1 Geology Department, Faculty of Science, Benha University, Benha, 13518 Egypt; 2 Geology Department, Faculty of Science, Suez University, Suez, 43518 Egypt; 3 Geology Department, Faculty of Science, Tanta University, Tanta, 31527 Egypt

**Keywords:** Solid Earth sciences, Geophysics, Tectonics

## Abstract

Integration of potential field- and structural data make it possible to trace surface and subsurface large-scale geological structures of the Egyptian Nubian Shield (ENS). Obtained results indicate that the Northern Eastern Desert (NED) of the ENS is dominated by relatively younger (c. 580 Ma) E–W and NE–SW trending extensional structures that were controlled by the evolution and retreat of the Cadomian Arc. Density of such extensional structures increases with depth as displayed by the potential data. The prevailing structural trends in the Central Eastern Desert (CED) are NW–SE and WNW–ESE. Both trends are highly prompted by the timing of deformation upon the Najd Fault System, and are themselves dissected by a relatively younger NE–SW shearing trend. Lineament density in the CED is subordinate for both subsurface and near surface structures. The South Eastern Desert exhibits compressional and extrusion-related structures of two main prominent trends; WNW-to-NW (to the western part) and the N-, NNE- to NE (to the eastern part). The previously mentioned Neoproterozoic trends are remarkably influenced by the Oligocene–Miocene Red Sea-Gulf of Suez rift related fractures in the vicinity of the rift shoulder. The remarkable change in trends and densities of structural trends, especially in the NED, is interpreted in terms of concealing of the older structures by the younger extensional structures which in turns reflect an N-ward progressive deformation in the entire ENS. Gravity data are more appropriate in delineating the structural trends compared to the magnetic data which are largely affected by lithological variations and/or alteration zones and magnetic mineralogy.

## Introduction

The parameters estimation of the causative bodies, i.e. its interment depth, lateral extent and amplitude of the physical feature variation, is the ultimate intention of a potential (magnetic and gravity) field (PF) survey^1^. Recently, PF data have become prime tools in regional tectonic prospects^2–4^, petroleum exploration^5,6^ and mineralization prospects^7–10^. Many techniques can be utilized in an automated mode to map the spatial distribution of physical parameters representing the sources in order to decipher the large scale structures. In the present study, the Logistic Total Horizontal Gradient (LTHG) filter^11^ was applied for delineating large-scale structures on both gravity and magnetic data of the ENS.

**Figure 1:**
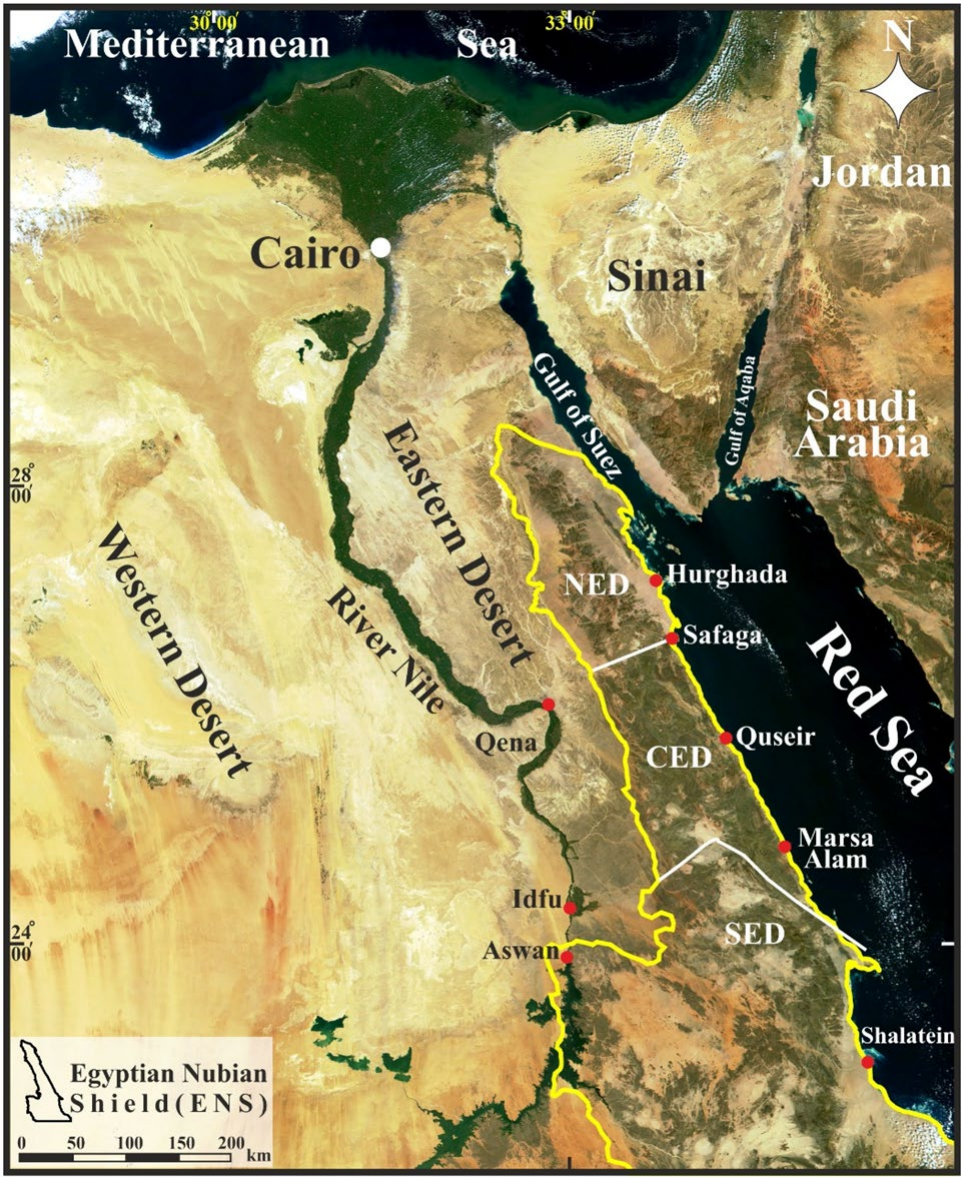
Landsat image showing the ENS and Northern Arabian Shield. Notice the tectonic provinces of the ENS; SED, CED and NED (Using ArcGIS v. 10.5 software; Powered by ESRI “Environmental Systems Research Institute”, www.esri.com).

**Figure 2:**
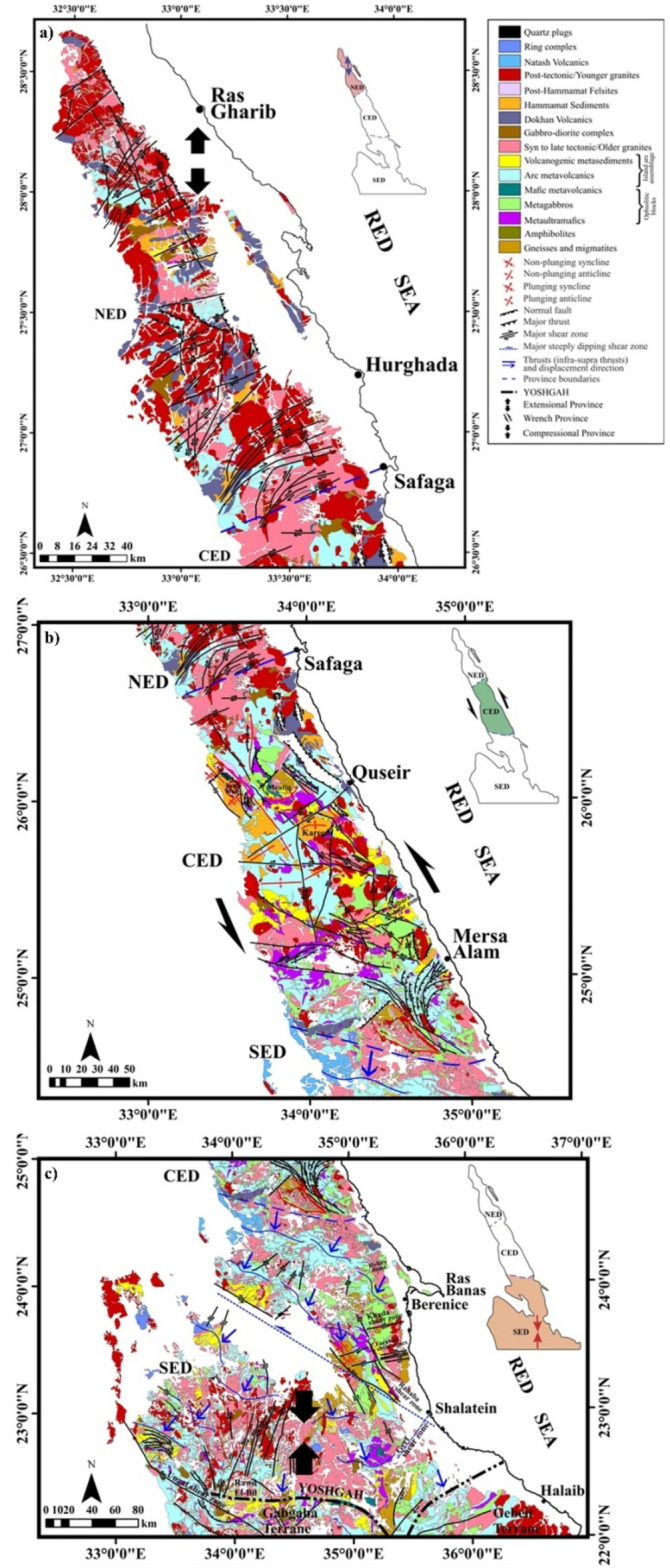
Detailed geological maps of (**a**) the NED, (**b**) CED and (**c**) SED constructed based on processing of remote sensing data and field/structural investigation (after^14^).

The ENS is exposed mainly over most of the Eastern Desert (ED) parallel to the coast of Gulf of Suez-Red Sea rift. It represents the northern continuation of the East African Orogen (EAO) and a prominent part in the Arabian-Nubian Shield (ANS, Fig. 1). The Neoproterozoic tectonic–metamorphic–magmatic evolution of the basement complex in the ED is highly correlated with that characterizing the ANS as both the shield and the ENS have been developed through the accretion–collision of arc-related tectonic terranes amalgamated along ophiolite-decorated suture zones which afterwards being deformed by post-accretionary structures represented mainly by major shear zones. The Neoproterozoic succession of the ENS is composed predominantly of ophiolites, metavolcanics and metasediments of volcanic arc affinity, gneissic-core complexes and associated post-collision Hammamat molasse sediments (Fig. 2a–c).

The general consensus that the ENS is liable to subdivision into three structural domains (North Eastern Desert, NED; Central Eastern Desert, CED; South Eastern Desert, SED)^12–14^ was merely premised on the conspicuous differences in basement lithology with little (if any) contribution to the ubiquitous changes of "internal" characteristic structures. The characteristic lithology of NED is the huge amount of granitod intrusions, and Dokhan Volcanics and Hammamat sediments, together with few exposures of island arc rocks (Fig. 2a). In contrast, the CED is distinguished by ophiolitic sequences and gneissic-cored domal-like structures and bounding shear zones^15^ (Fig. 2b). The SED is defined by high-grade gneisses and metasedimentary schists, and has the noteworthy Alaqi-Heiani Suture and the large Gerf ophiolitic nappe (Fig. 2c). The boundaries between the previous structural provinces were regarded as tectonic hinges or megashears; Qena-Safaga Shear Zone between NED and CED, Idfu-Mersa Alam Shear Zone (Nugrus-Shait Shear Zone^16^ between CED and SED).

Although the two-tier “suprastructure–infrastructure” orogenic model^17,18^ and the previously mentioned threefold classification of the ENS into three structural provinces have widely accepted, claim for further studies based on geophysical methods (seismic, gravity and magnetic) in order to detect the major subsurface tectonic features and to explore the surface structures with depth is highly recommended^19^. In addition, recent comprehensive regional structural/tectonic studies and associated isotopic data^14,18–23^ have contributed to the structural setting and tectonic evolution of the ANS and ENS, as well as they encourage new reliable approaches for deciphering the problematic issues and examine the validity of the applied models. In this context, the present study is the first attempt to intigrate structural and potential data (gravity and magnetic) for deciphering the large-scale structures of the Pan-African belt in the ENS. Field-structural data with the aid of lithological and structural mapping can easily visualize the major surface tectonic structures. Furthermore, the potential data are a powerful tool for detecting the near surface and also the subsurface deep structures. Integration of both studies and/or methods will facilitate accurate investigation of the tectonic boundaries between structural provinces and allow a sort of correlation between surface and subsurface structures in terms of their trends, density and penetrability.

## Potential data enhancement and results

### Aeromagnetic data

**Figure 3:**
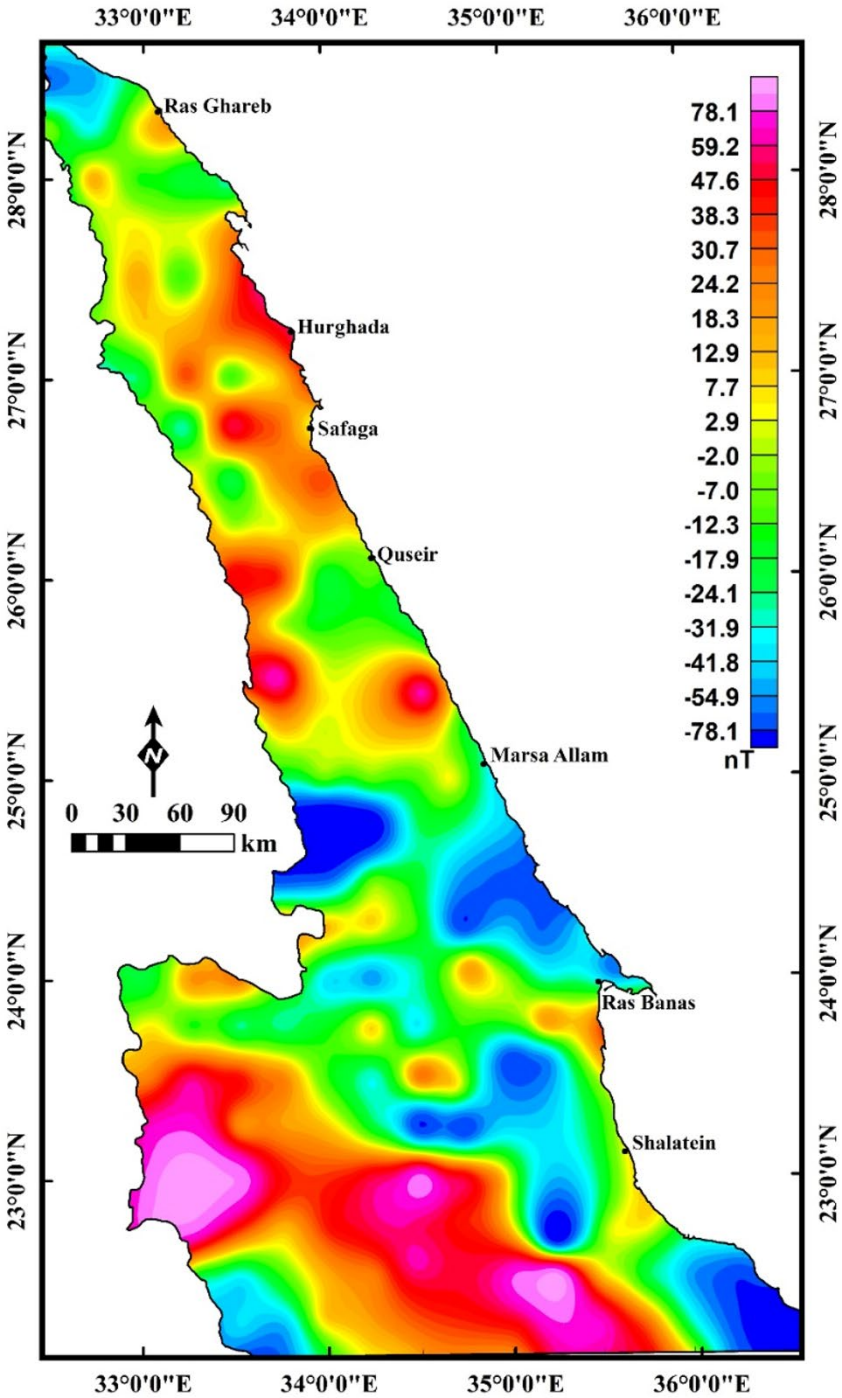
Magnetic anomaly (MA) map used in the present study (Using  Geosoft Oasis Montaj 2015 v. 8.3.3 software, https://www.seequent.com/help-support/oasis-montaj/).

**Figure 4:**
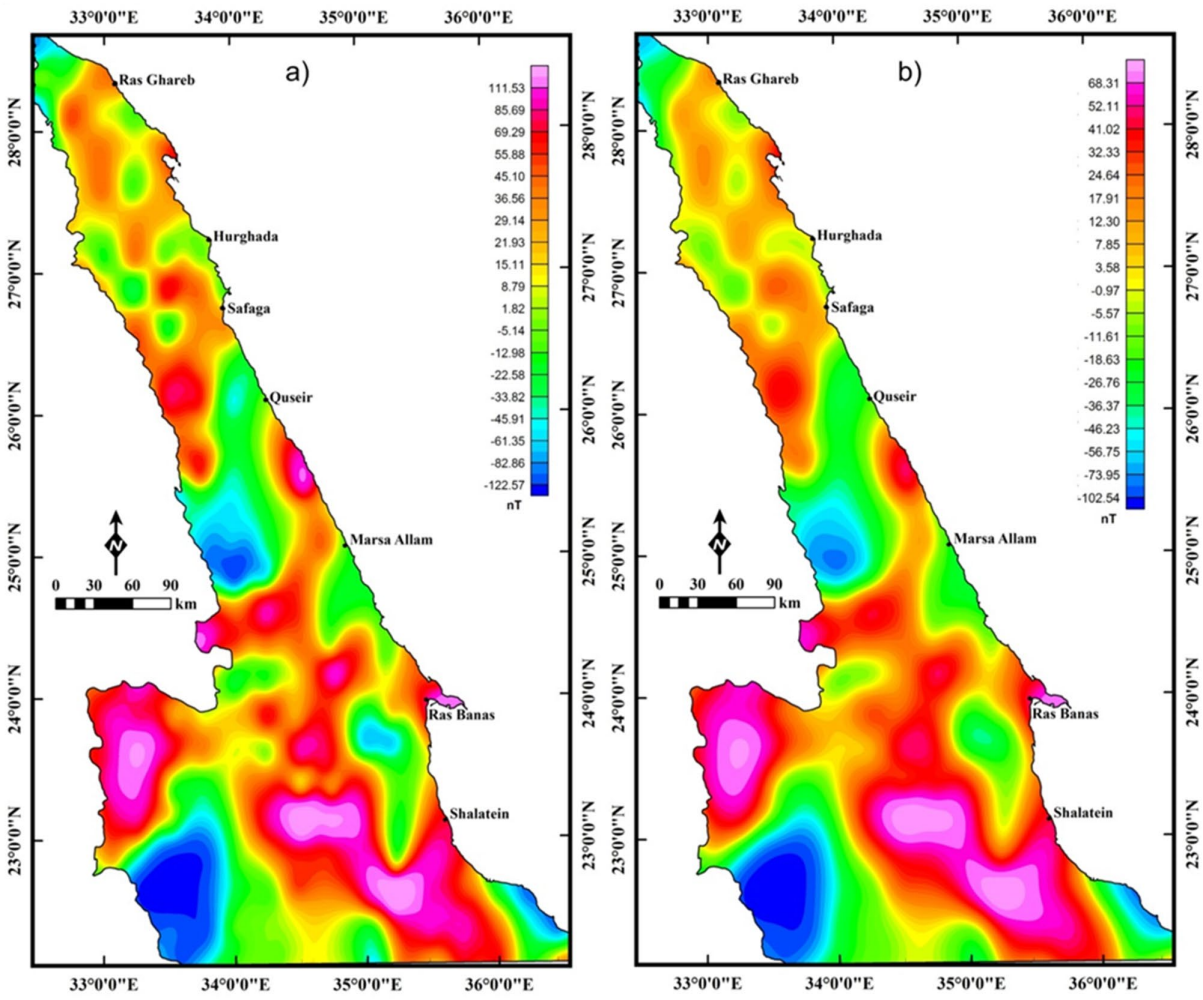
(**a**) Reduced to the pole (RTP) magnetic anomaly (MA) map; (**b**) the upward continuation (altitude of 8 km) map of the RTP data (ENS), (Using  Geosoft Oasis Montaj 2015 v. 8.3.3 software, https://www.seequent.com/help-support/oasis-montaj/).

The magnetic anomaly (MA) data (Fig. 3) were acquired from the Co-operative Institute for Research in Environmental Sciences (CIRES) and Earth Magnetic Anomaly Grid 2-arcminute resolution dataset (EMAG2), gathered from a combined marine, airborne, and satellite data^24^. The magnetic anomalies (MAs) of the ENS show variation in magnetization values ranging from less than -200 to more than 150 nT. These abnormalities are linked to the changes in either topographic relief or the distribution of geologic rock units. The anomalies are displayed as diverse color spectra where the magnetic highs (reddish brown) are related to the presence of high ferromagnetic (basic) rocks and the magnetic lows (blue) indicate acidic or metasedimentary rocks. The magnetic data was reduced using the reduced to pole “RTP” method^25^ (Fig. 4a). The RTP map (Fig. 4a) is characterized by high magnetic responses in the southern ENS due to the presence of mafic–ultramafic rock units and complex history of deformation and tectonism and magnetization values tend to decrease towards the north. The RTP map in Fig. 4a is upward-continued (UPC)^26^ to an altitude of 8 km (4 km depth^3^, Fig. 4b). The map acquired from the utilization of UPC (Fig. 4b) exhibits the area to be more homogeneous.

**Figure 5:**
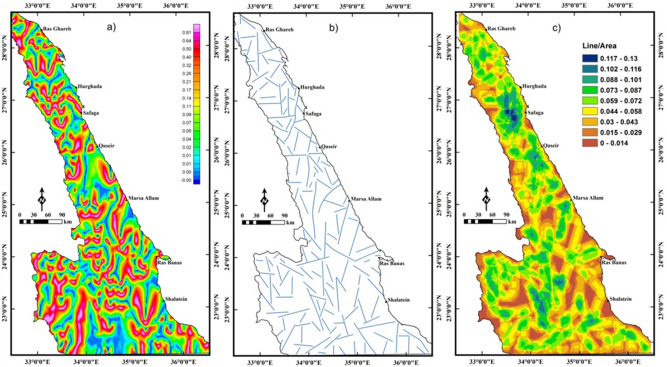
(**a**) LTHG-RTP map; (**b**) the interpreted structures from the LTHG-RTP map; and (**c**) LTHG-RTP lineament density map (Using  Geosoft Oasis Montaj 2015 v. 8.3.3 software, https://www.seequent.com/help-support/oasis-montaj/ and ArcGIS v. 10.5 software; Powered by ESRI “Environmental Systems Research Institute”, www.esri.com).

The LTHG filter is applied to RTP data (Fig. 4a) for deciphering the large-scale structures of the Pan-African belt in the ENS. Faults and boundaries in the LTHG-RTP map (Fig. 5a) are interpreted as a structural map of the study area (Fig. 5b). This map shows the following main tectonic trends: NW, WNW, NE, E–W, NNE, ENE and N–S. A lineament density map (Fig. 5c) constructed from the structural map in Fig. 5b revealed that the southern ENS is highly deformed than the central and northern parts.

**Figure 6:**
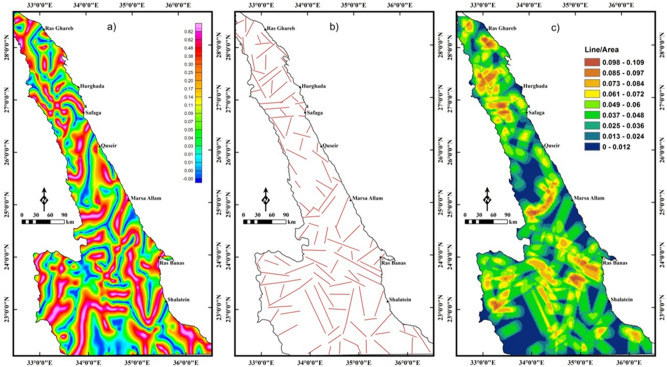
(**a**) LTHG-UPC-RTP map; (**b**) the interpreted structures from the LTHG-UPC-RTP map; and (**c**) LTHG-UPC-RTP lineament density map (Using  Geosoft Oasis Montaj 2015 v. 8.3.3 software, https://www.seequent.com/help-support/oasis-montaj/ and ArcGIS v. 10.5 software; Powered by ESRI “Environmental Systems Research Institute”, www.esri.com).

To map the deep structures, the LTHG is applied to the UPC-RTP map (Fig. 6a). The deep structures (4 km depth) in the LTHG-UPC-RTP map (Fig. 6a) are interpreted and represented in a structural map (Fig. 6b). Figure 6b shows that the southern ENS is mainly dominated by the NW, WNW and NE trends, with some traces of the NE and E–W directions. The NW, NE and WNW are dominant in the central ENS, while the E–W, NW and NNE with small traces of the N–S ones are the main directions that affecting the northern ENS. The lineament density map (Fig. 6c) of the LTHG-UPC-RTP represents that the southern ENS is still the highly deformed part. Moreover, at depth of 4 km, the northern ENS has a higher lineament density than the central part and some tracts in the southern part (i.e., in the higher levels of the NED the major structures are disturbed and concealed by the voluminous intrusions of older and younger granitoids). Figures 5b,6b show that the NW and WNW directions are scattered by the NE direction indicating that the NE trend is the younger one.

### Gravity data

**Figure 7:**
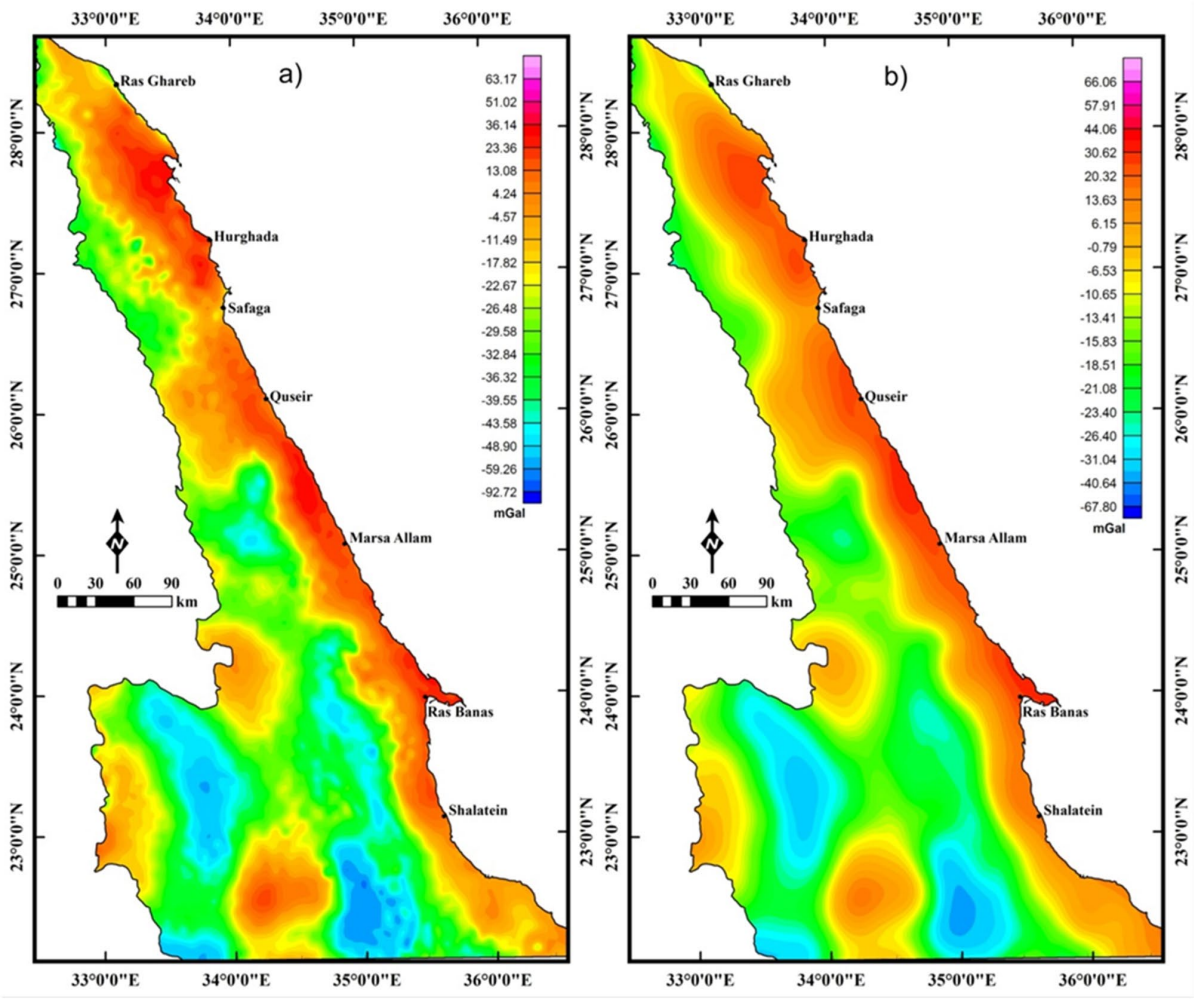
The Bouguer gravity (BG) map (**a**) and the upward continuation map (altitude of 8 km) (**b**) of the BG data of ENS (Using  Geosoft Oasis Montaj 2015 v. 8.3.3 software, https://www.seequent.com/help-support/oasis-montaj/).

The employed gravity data in the present study are the Bouguer gravity (BG) anomalies derived from the World Gravity Model (WGM) 2012^27^. The BG anomaly map (Fig. 7) displays anomalies that reflect shorter- and longer-wavelengths. The long-wavelength anomalies are usually related to deep and wide density sources and the anomalies of short-wavelengths are commonly caused by tiny width and shallow density sources. The BG anomaly map of the study area (Fig. 7a) shows an increase in density values (positive anomalies) towards the east due to the oceanic crust areas in the Red Sea. This BG map is upward-continued (UPC)^26^ to an altitude of 8 km (4 km depth^3^, Fig. 7b). The map acquired from the utilization of UPC (Fig. 7b) exhibits the area to be more homogeneous in the density values.

**Figure 8:**
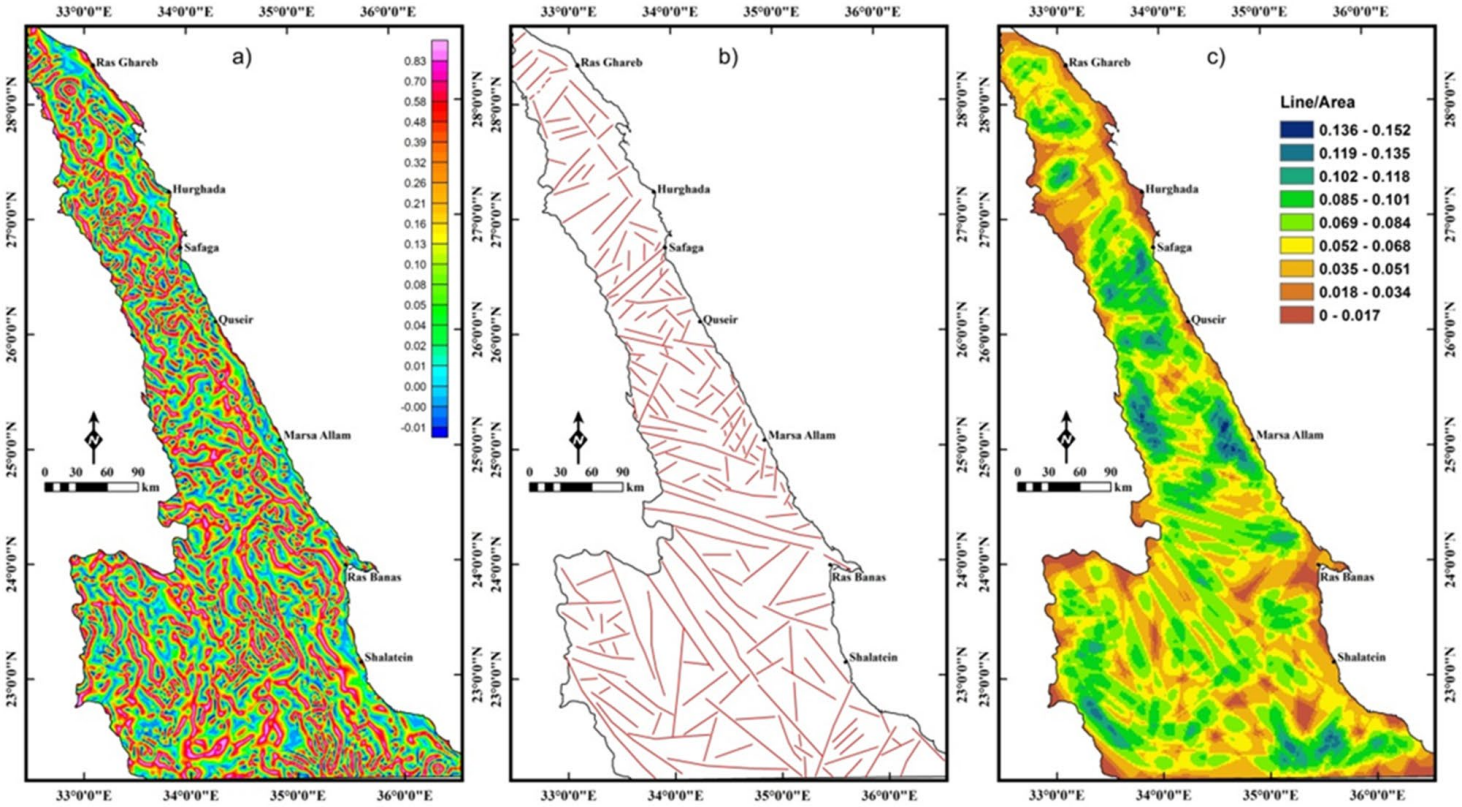
(**a**) LTHG-BG map, (**b**) the interpreted structures from the LTHG-BG map, and (**c**) LTHG-BG lineament density map (Using  Geosoft Oasis Montaj 2015 v. 8.3.3 software, https://www.seequent.com/help-support/oasis-montaj/ and ArcGIS v. 10.5 software; Powered by ESRI “Environmental Systems Research Institute”, www.esri.com).

The LTHG filter is utilized to BG data (Fig. 7a) for revealing the tectonic structures of the Pan-African belt in the NED. The edges and faults in the LTHG-BG map (Fig. 8a) are interpreted in a structural lineament map (Fig. 8b). The structural map (Fig. 8b) shows that the main fault trends affecting the southern ENS oriented NW, WNW and NE, while the central ENS is dominated by the WNW to NW and NE fault trends. The NE, NW and E–W are the main structural trends affecting the northern ENS. A lineament density map (Fig. 8c) is generated from the structural map in Fig. 8b and outlined that the southern ENS is highly deformed than the central and northern parts.

**Figure 9:**
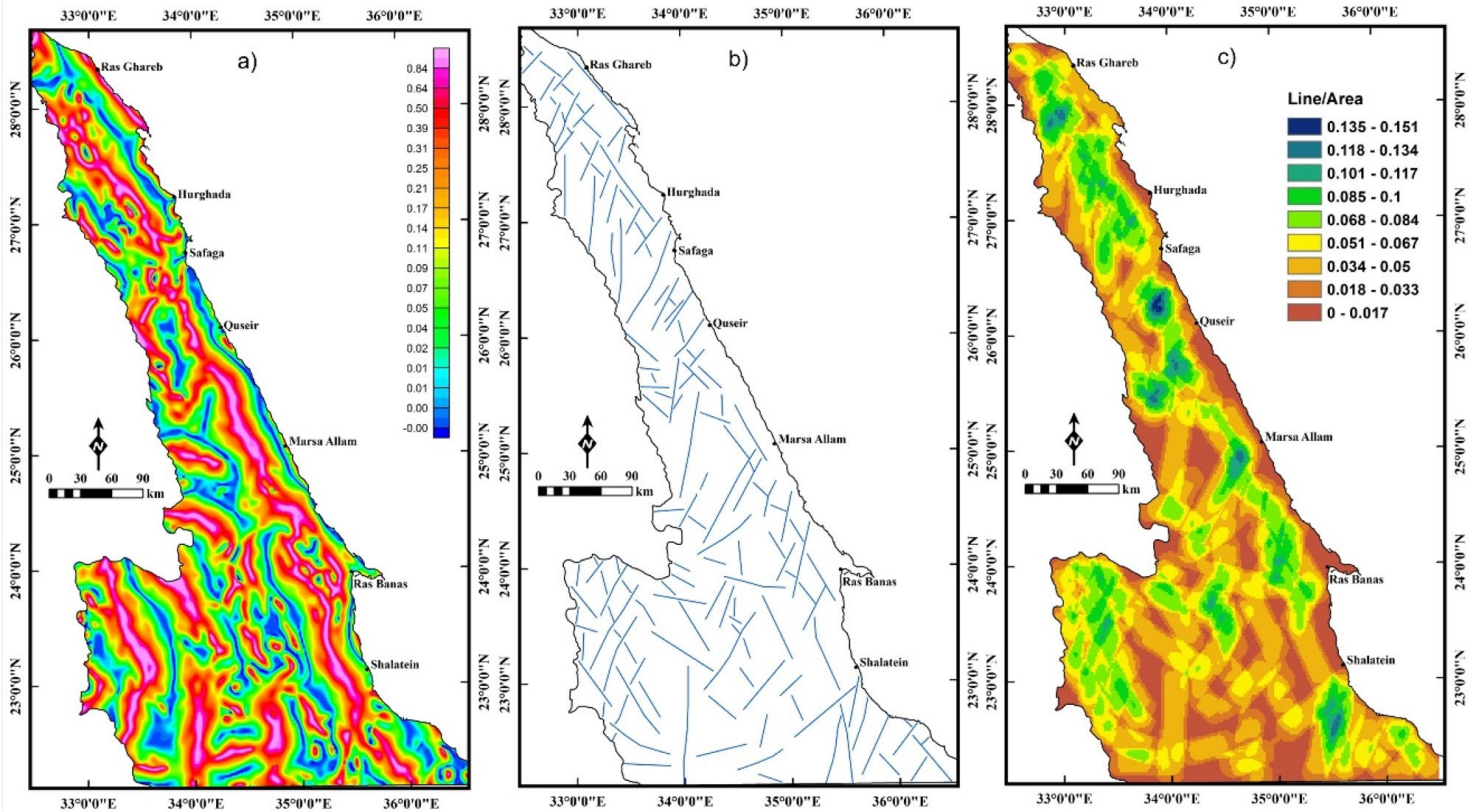
(**a**) LTHG-UPC-BG map, (**b**) the interpreted structures from the LTHG-UPC-BG map, and (**c**) LTHG-UPC-BG lineament density map (Using  Geosoft Oasis Montaj 2015 v. 8.3.3 software, https://www.seequent.com/help-support/oasis-montaj/ and ArcGIS v. 10.5 software; Powered by ESRI “Environmental Systems Research Institute”, www.esri.com).

As in magnetic data, the LTHG is applied to the UPC-BG map in Fig. 7b to map deep structures. The deep structures (4 km depth) in the LTHG-UPC-BG map (Fig. 9a) are outlined in a structural map (Fig. 9b). Figure 9b shows that the southern ENS is mainly dominated by the NW, WNW and NE trends. The NW, NE and NNE trends are dominant in the central and northern ENS. The lineament density map (Fig. 9c) of the LTHG-UPC-BG represents that the southern ENS is the highly deformed part of the ENS. Figures 8b,9b show clearly that the NW and WNW tectonic trends have been truncated by the NE direction in many parts of the ENS confirming that the NE trend is the younger trend.

## Discussion of the potential data results in relation to the structural setting of the ENS

The Logistic Total Horizontal Gradient (LTHG) filter^11^ is applied for detecting large-scale structures on both the gravity and magnetic data, giving an interesting and in practice realistic regional overview on the near surface as well as deep tectonic structures and their structural trends. The obtained structural maps and associated interpreted structures (mainly faults and tectonic boundaries between the different litho-units represented on maps in Fig. 2a–c) enabled a significant window on the nature of crustal levels underneath the ENS.

**Figure 10:**
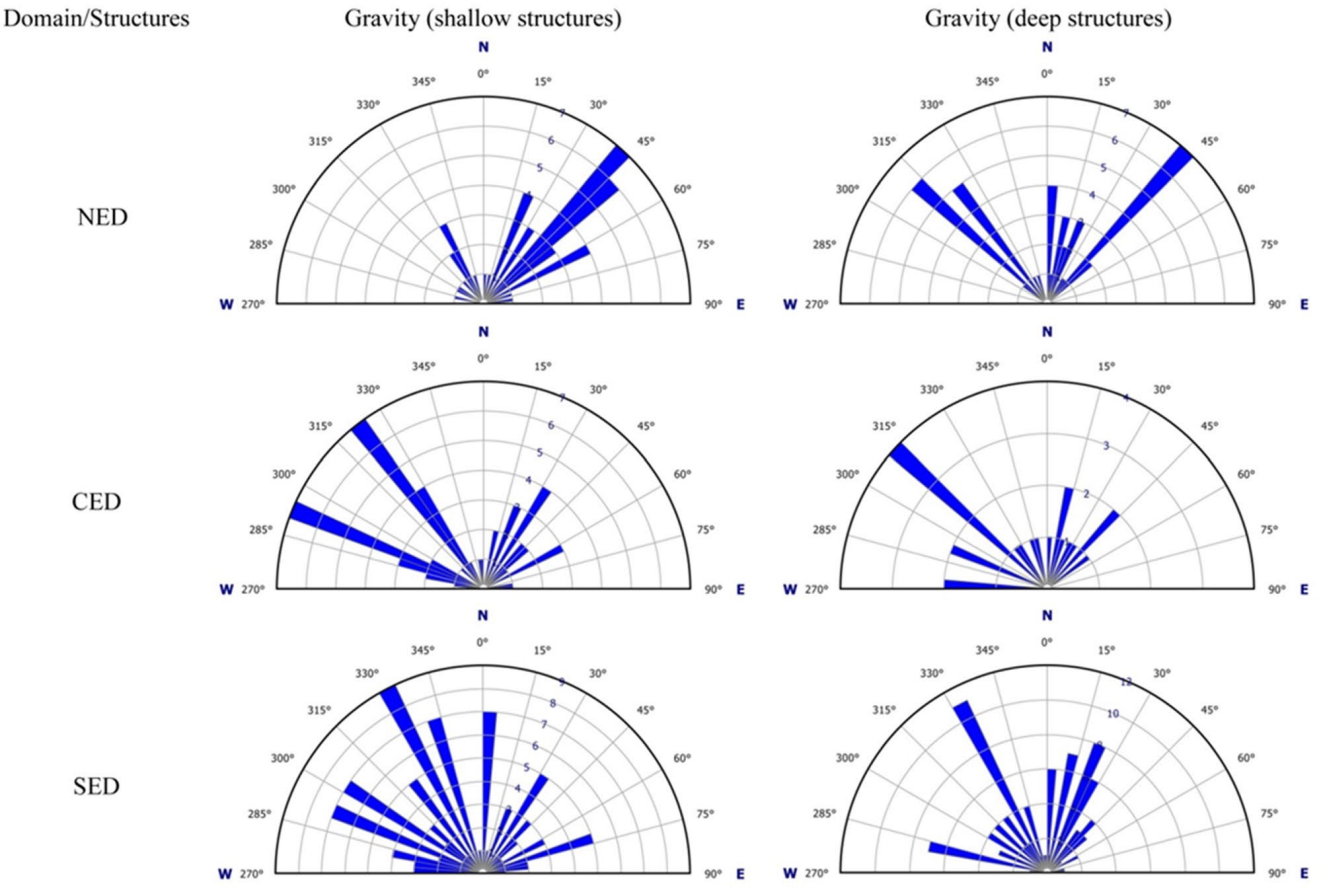
Rose diagrams showing the structural lineaments interpreted from gravity data including both shallow and deep structures of the NED, CED and SED tectonic provinces.

**Figure 11:**
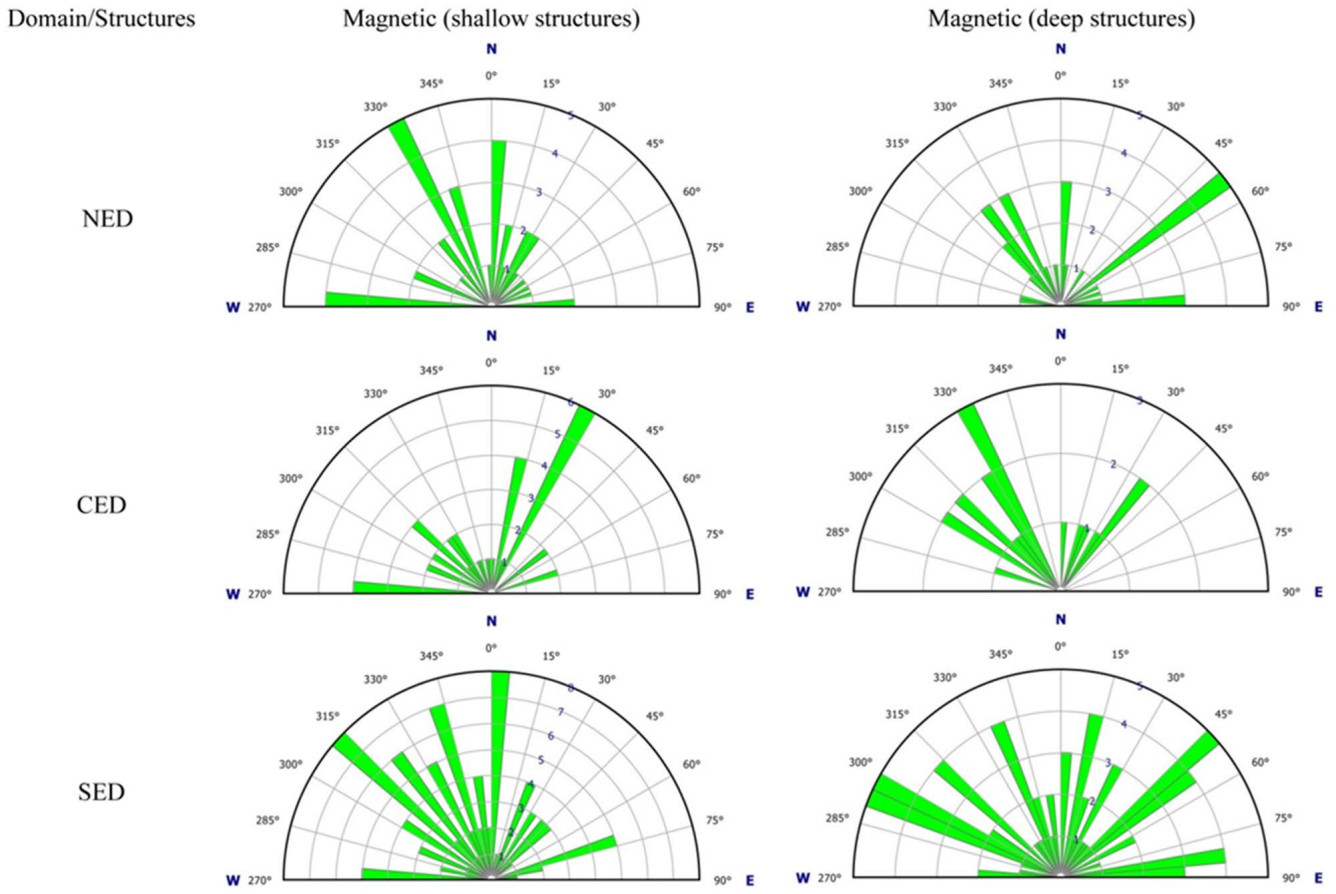
Rose diagrams showing the structural lineaments interpreted from aeromagnetic data including both shallow and deep structures of the NED, CED and SED tectonic provinces.

Gravity and magnetic data are shortly interpreted during the presentation and enhancement of the potential data (Figs. 3,4,5,6,7,8,9) depending on the geophysical concepts and basics. Here, further (detailed) structural interpretation for the aeromagnetic and gravity results, particularly the tectonic trends of the interpreted structures and their densities, was contributed. The tectonic trends of the interpreted lineaments/structures (mainly faults and shear zones) detected from the LTHG-RTP, LTHG-UPC-RTP, LTHG-BG and LTHG-UPC-BG maps are graphically represented using rose diagrams (Figs. 10,11). For simplification, the ENS in the Eastern Desert subdivided into three structural provinces (NED, CED and SED) following the traditional tripartite classification^12,13^. At first glimpse, a high degree or even complete matching with little deviations can be recognized (Figs. 5b,6b,8b,9b) between the main trends within the three provinces for both the gravity and magnetic data (shallow and deep). However, the tectonic trends interpreted from the gravity maps have the priority as they accurately depict the changes in tectonic trends between structural provinces and in the same time the shallow interpreted structures are highly fitted with the geological and structural setting of the concerned provinces. Regarding the northern ENS, the roses of gravity maps (Fig. 10) show the N43°–45° as major tectonic trend and N320°–330° as secondary one. For the magnetic maps (Fig. 11), the main tectonic trends interpreted from deep magnetic data are in complete harmony with those represented from the gravity maps, while the rose diagrams of shallow magnetic data demonstrates other tectonic trends; N0°–5°, N330° and N270°–275°). In the central ENS the representation of the interpreted structures on rose diagram showing high matching level between the gravity (both shallow and deep) and deep magnetic data (N290°–N300° as major trends and N15°–40° as secondary trends), while the shallow magnetic data yields opposite directions (N15°–30° as main trend and N270°–275° to N315°–330° as secondary trends). In contrast to the northern ENS and central ENS, the southern ENS seems to be a complex structural province with several tectonic trends where the representation of magnetic and gravity data shows different behavior. Here, the shallow gravity and magnetic structures look identical in their represented trends, but the deep structures of both data have two to three subordinate tectonic trends; deep gravity structures have N330°–335°, N10°–30° and N280°–285° trends (from major to minor), while the magnetic deep structures have tectonic trends in N340°, N310°–315°, N10°–15°, N80°–90°, N45°–55° and N290°–300° directions (from minor to major).

The structural and tectonic interpretation of the previously represented structures can be discussed in terms of the geology, structural setting and tectonic evolution of the ENS as a contiguous part of the ANS. Three aspects could be addressed to unravel the apparent changes in structural trends from a province to another and also from shallow to deep crustal levels along the whole ENS, (1) the structural setting of each province and the associated basement assemblage, (2) the kind/nature of the potential data used, and (3) as the structural provinces in the ED are juxtaposed along major structural discontinuities (megashears), the investigation of the interpreted structures to check the exact position of such boundaries is highly recommended here. These aspects will be discussed for each structural province in the next subsections, followed by some critical concluding points.

### NED structural province

**Figure 12:**
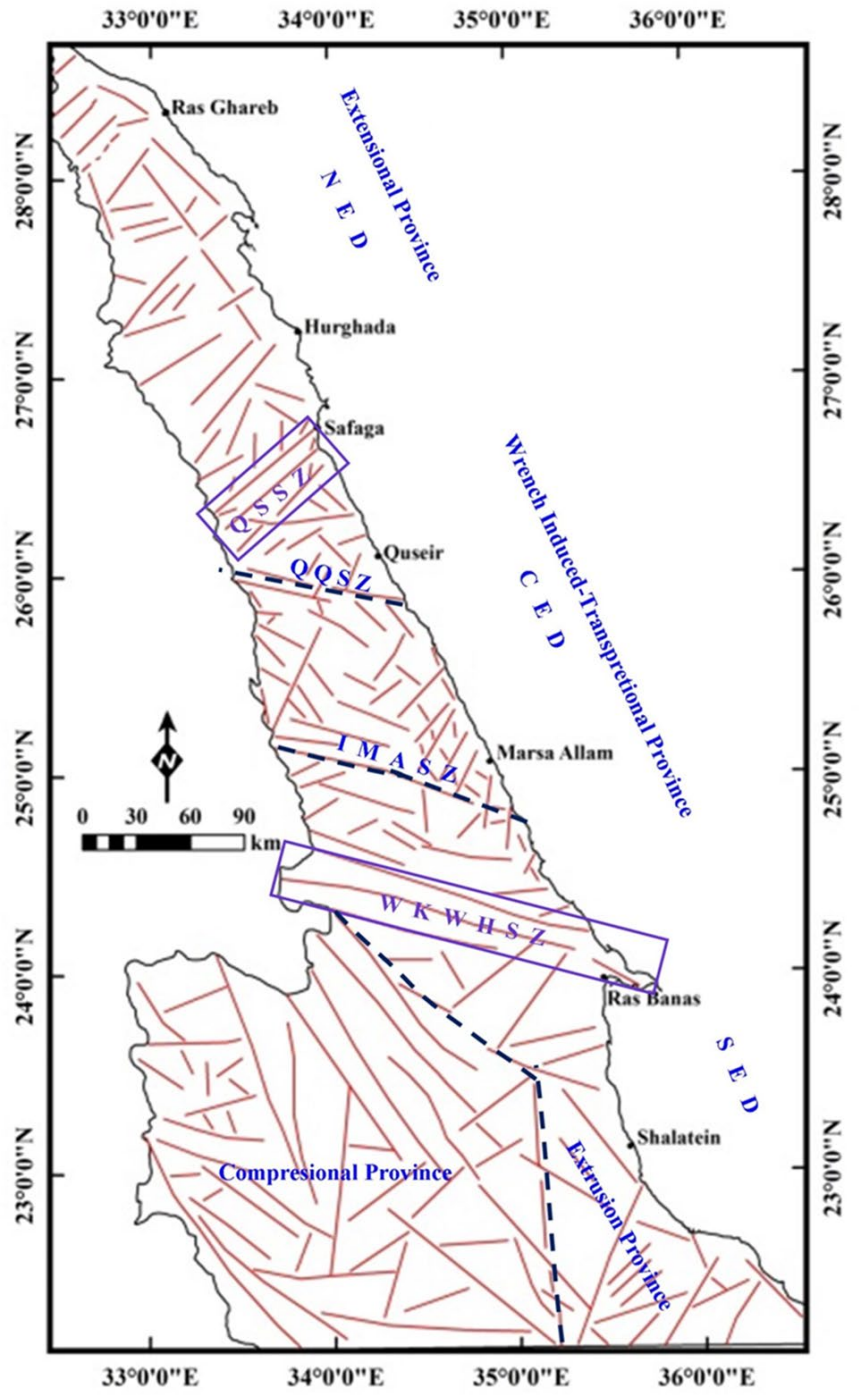
The proposed tectonic provinces; extensional province (NED), wrench induced-transpresional province (CED) and compressional/extrusional province (SED), with their internal and external tectonic boundaries drawn on the structural map produced from gravity data. QSSZ: Qena-Safaga Shear Zone; QQSZ: Qift-Quseir Shear Zone; IMASZ: Idfu-Marsa Alam Shear Zone; WKWHSZ: Wadi Kharit–Wadi Hodein Shear Zone; NED: North Eastern Desert; CED: Central Eastern Desert; SED: South Eastern Desert.

The NED structural province is largely occupied by granites with subordinate amounts of Hammamat-type sediments and a few exposures of arc-related metavolcanic and metasedimentary associations (Fig. 2a). This province is proposed here to be extended farther to the northern tip of the ENS, and separated from the CED province by a broad zone/belt of NE–SW trending faults extended southward from the Qena-Safaga shear zone until the NE–SW oriented structural fabric gradually intersected with the NW–SE trending faults characterizing the CED province to the north-northwest of Quseir (Fig. 12). In this study, such belt will be considered as the tectonic boundary between the NED and CED structural provinces. The dextral shear criteria have been encountered some kilometers to the west of the Safaga City on the Red Sea coast^13,28^. The formation of Qena-Bend is presumably resulted from the reactivation of the Qena-Safaga Shear Zone during the Tertiary time^13^ and the open fractures and ongoing high level seismicity are also consequences of the present day activity on the Qena-Safaga Shear Zone^29^.

**Figure 13:**
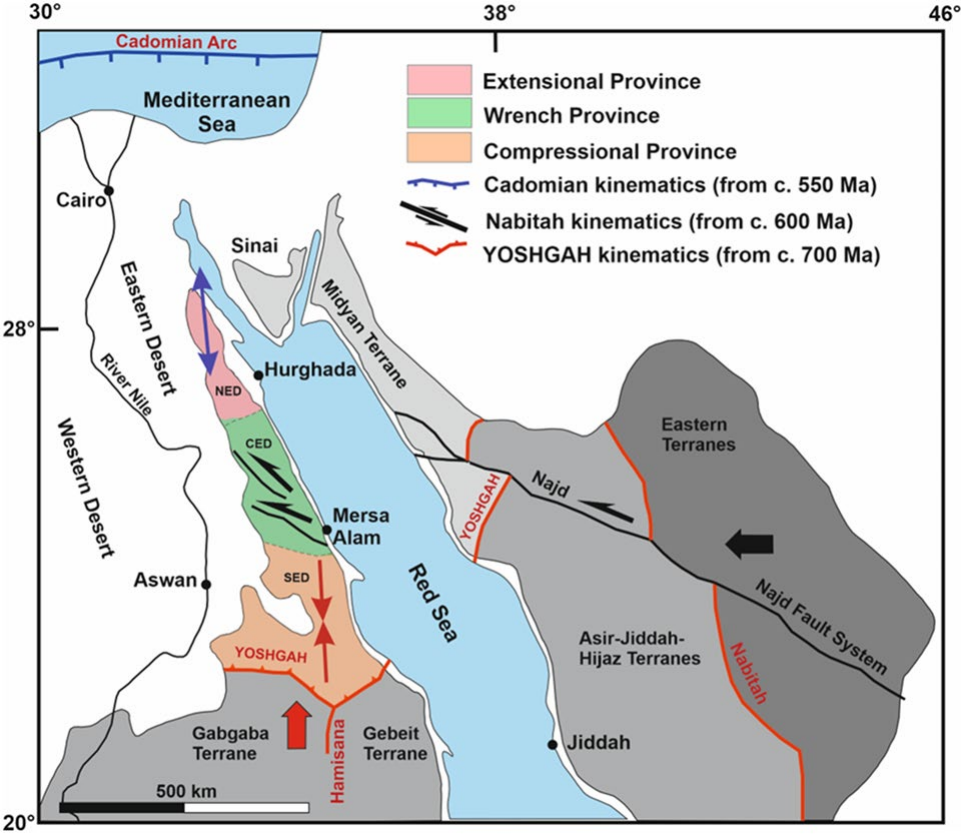
Plate tectonic situation and structural provinces related to different plate kinematics. The earliest event, convergence, and collision between Gabgaba-Gebeit terranes and the Eastern Desert terrane along the YOSHGAH suture released north–south compression (thick red arrow) and related north and south directed thrusts (red double arrow) in the southern compressional province. West–east convergence (thick black arrow) associated with Nabitah Orogeny caused modification of suture and W–E compression in the Hamisana Belt. In the northern portion, this motion is partitioned into large sinistral strike-slip systems of the Najd Fault System leading to transcurrent motion within the CED wrench province (black half arrows). The retreat of the northern Cadomian arc enabled extension in the northern NED extensional province (blue double arrow) (after^14^).

Beside the voluminous intrusions of older and younger granitoids, the NED structural province is characterized by expanded fields of E–W oriented dike swarms which indicate the extensional origin or nature of this province. The E–W and NE–SW structures are dominated in the NED province, although the NW–SE tectonic trend is delineated on rose diagrams (Figs. 10,11). The existence of NW–SE trending faults can be easily interpreted in terms of the Oligocene–Miocene opening of the Red Sea-Gulf of Suez rift, where many major NW–SE normal faults bounding the western shoulder of the rift (Figs. 8b,9b). In this province, the density of structures increases with depth (4 km) in both gravity and magnetic upward continuation maps (Figs. 6b,9b) which indicates that in the upper crustal levels of the NED the main structures are disturbed and concealed by the voluminous intrusions of older and younger granitoids and the extensive post-granitic dyke swarms. Such setting could also reveal that the tectonic regime was changed from compressional and transpressional as in the southern and central provinces (respectively) to extensional during the latest tectonic events of the Pan-African orogeny in Late Neoproterozoic (Ediacaran time) (Fig. 13).

### CED structural province

The CED structural province is composed mainly of suprastructural rocks (ophiolites and island arc assemblages), in conjunction with the shear zone-related gneissic domes (e.g. Migif-Hafafit, Meatiq, Sibai and Shalul domes)^15,30,31^ and associated Hammamat basins (e.g. Karim, Hammamat and Zeidun basins) (Fig. 2b). As a matter of fact, the CED occupied the core of the widely known Najd Shear Corridor within the ED where it proposed to be delineated by the Wadi Kharit–Wadi Hodein Shear Zone to the southwest and the Qena-Safaga Shear Zone to the north. This region comprises a variety of wrench-related structures in the southern reaches which are gradually upgraded northwards into conspicuous transpressional structures (Fig. 13). The CED structural province includes several major shear zones, such as the E–W oriented Idfu-Mersa Alam Shear Zone, Najd-related NW-oriented Nugrus Shear Zone and Atalla Shear Zone. In the area between Idfu-Mesa Alam- and Qena-Safaga Shear Zones, the transpressional and extensional structures are dominated and interplayed. These structures are originally attributed to the crustal-scale Najd Fault System which characterized by brittle to semi-ductile shear zones represented on various scales ranging from single faults to mountainous sinisterly mega-shear zones. The existence of a wide variety of deformational structures led to the grading in metamorphic facies from low- and medium-grade in suprastructural rock units to high-grade in the core of gneissic domes. In addition, the later intrusion of syn- to late-tectonic and post-tectonic granites facilitated the distribution of mineralized zones. According to the previous structural and tectonic setting of the CED, the main direction of faults and major structures is NW–SE and WNW–ESE (Figs. 10,11). The NW- and WNW- oriented structures are truncated by the NE–SE oriented faults indicating the NE–SW tectonic trend as the younger trend (the secondary trend in Fig. 10). Furthermore, the density of the interpreted structures in the CED is relatively higher than that in the NED. The tectonic boundary between the CED and SED is a matter of debate where the Idfu-Mersa Alam Shear Zone is traditionally agreed, and the Nugrus-Shait Shear Zone is highly recommended^16^. According to the present study results the Wadi Kharit–Wadi Hodein Shear Zone is not excluded because the structural trends immediately to the south of such belt are bifurcated into two main trends WNW–ESE and NE–SW which are characterized as the major structural trends in the SED structural province (Fig. 12).

### SED structural province

The SED structural province (Fig. 2c) formed of a mosaic of complex areas of gneisses and migmatites, highly schistose metasediments and deformed older granites (granodiorites and tonalites)^13,32^. This province is characterized by the earlier compressional and extrusional structures (Fig. 12). Further in the extreme southwestern corner of the SED, the prominent Wadi Allaqi Suture Zone do exist, where the older phase of N–S compression has been occurred ∼ 750–660 between the SED and Gabgaba terranes (Fig. 13). The S- to SW propagated thrusts and thrust-related folds are very characteristic for such region. The main structural trends at this area are WNW–ESE to NW–SE (Figs. 8b,9b). Eastwards, the Wadi Allaqi Suture Zone is abruptly truncated by the extrusion-induced structures (Fig. 12). The northward expulsion/extrusion of the ANS from the Mozambique Belt^33^ was resulted in the northward lateral movement on the Hamisana Shear Zone and the extrusion/escape of the ENS relative to the Saharan Metacraton (Fig. 13). The tectonic stacking of the ophiolitic rocks at Gabal Gerf and the formation of some major shear zones, such as Wadi Kharit–Wadi Hodein Shear Zone and the other NW–SE Najd-related NW–SE shear zones are the later consequences of such tectonic movement. According to the structural studies and the interpreted structures in the present study, the tectonic trends of the extrusion structures are N–S, NNE–SSW and NE–SW which have been represented on the rose diagrams (Fig. 10,11). Concerning the structural and lineament density maps extracted from the magnetic and gravity data, the density of the interpreted structures for the SED province is absolutely the highest one in both deep and shallow crustal levels (Figs. 5c,6c,8c,9c). This observation is in complete harmony with the previously discussed structural and tectonic setting, and evidently concluding that the southernmost province (SED) from the ENS is the oldest and most deformed terranes.

## Methodologies and software-used

**Figure 14:**
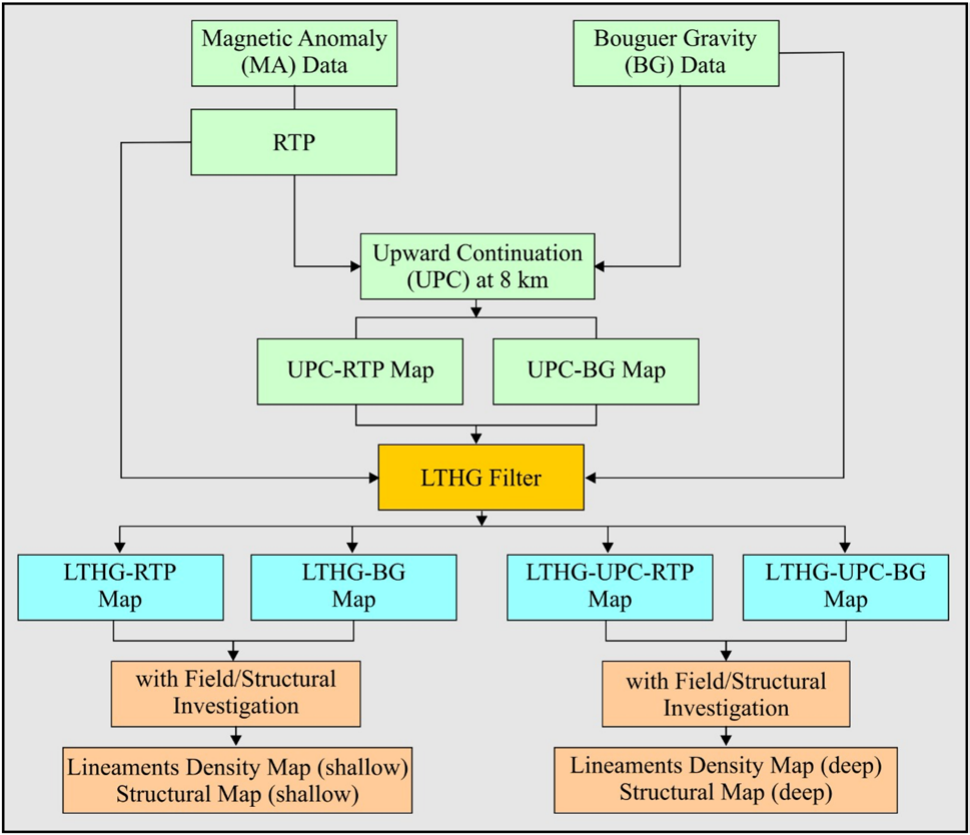
Flowchart showing the data analyses and methods used in the present study.

The theoretical and mathematical bases of the applied enhancements for detection of PF data of the large structures in the study area will be illustrated in this section (Fig. 14). The LTHG approach represents the ratio of the first vertical and the total gradients of horizontal derivatives (HD) that give noticeable tiny and large amplitude boundaries together. The purpose of including this method is that the numerical logistic function produces a sigmoidal curve. Its S-appearance is extremely relevant to that of the arctangent equation that is ordinarily applied to recognize edges and sides of potential fields^11^.

1 $$LTHG = \left[ {1 + exp\left\{ { - \frac{{\frac{\partial THG}{{\partial z}}}}{{\sqrt {\left( {\frac{\partial THG}{{\partial x}}} \right)^{2} + \left( {\frac{\partial THG}{{\partial y}}} \right)^{2} } }}} \right\}} \right]^{ - \alpha }$$

The positive constant (α) manages the potential of the LTHG and defined by the researcher. Experiment returns on synthetic data authenticate that the most substantial returns were generally obtained when employing α = 2–10. THG is the total horizontal amplitude, $\partial x$ and $\partial y$ are the HD in x and y directions, and $\partial z$ is the vertical derivative of the field.

The LTHG technique is one of the most powerful enhancements for recognizing the boundaries of potential (magnetic and gravity) field sources giving sharp and accurate structural mapping^11,34^. The magnetic and gravity are upward continued (UPC)^35^ to an altitude of 8 km (4 km depth). The LTHG filter will be applied to both original and upward continued data of the study area to map shallow and deep structures, respectively. The Surfer (v. 13), Geosoft (v. 6.4) and ArcGIS (v. 10.4) are used to enhance, interpret and represent the potential data of the study area.

## Conclusions

The following points can be concluded from the interpretation of the processed and interpreted potential and structural data:

1. The Pan-African belt in the ED of Egypt can be subdivided into at least three major structural/tectonic provinces based on the conspicuous change in basement lithology, structural trends and tectonic regime.
2. The three provinces are SED, CED and NED from older to younger and also from south to north. These provinces are separated by major tectonic discontinuities (broad shear belts) conformed with the Qena-Safaga Shear Belt between the NED and CED, and most probably the Wadi-Kharit-Wadi Hodein Shear Belt between the CED and SED. The Idfu-Mersa Alam and Nugrus-Shait Shear Zones are less discriminated among the interpreted shallow and deep structures.
3. The SED is characterized by prominent compressional and estimated extrusionalstructures. The Allaqi Suture Zone and the Hamisana Shear Zone are directly related to these tectonic movements, respectively. It is considered here as the oldest (highly deformed) structural province and of the highest lineament density among the three provinces. The main tectonic trends here are WNW-to-NW in the western part of the province and the N- , NNE- to NE in the eastern parts.
4. The CED is characterized by transpressional and extensional structures which kinematically akin to the Najd Orogeny. The main tectonic trends are NW–SE and WNW–ESE, while the NE–SW tectonic trend is being younger and dissecting the Najd shear trend in places. The lineament density of that province is subordinate for both subsurface and near surface structures. The age of the tectonic structures within this large province is highly prompted by the timing of deformation upon the Najd Fault System.
5. The NED is characterized by extensional structures with E–W and NE–SW main tectonic trends. The density of structures is low at surface or near surface and increases gradually with depth as indicated from the magnetic and gravity data. The age of the structures in this province is intimately related to the latest tectonic events affecting the Gondwanalands in the late Ediacaran time (younger than 550 Ma)
6. The Pan-African tectonic structures are highly affected by the NW–SE (N25°–30°W) oriented faults which are dated back to the opening of the Red Sea-Gulf of Suez during the Oligocene–Miocene time, particularly at the eastern border of the shield along the western shoulder of the Red Sea.
7. There is a remarkable change in trend and density of interpreted structures with depth, especially in the NED where the older structures are concealed by the younger extensional deformation which indicating that the crustal evolution of the belt was operated progressively from south to north.
8. The gravity data are most suitable for depicting/delineating the tectonic trends accurately, and although the interpreted structures are moderately to highly correlated with those detected from the magnetic data, the interpreted structures from magnetic data are largely affected by the strike change in the lithology, alteration zones and magnetic mineralogy.

## Data Availability

The datasets used and/or analyzed during the current study are available from the corresponding author on reasonable request.
